# Comprehensive understanding of the mutant ‘giant’ *Arthrospira platensis* developed via ultraviolet mutagenesis

**DOI:** 10.3389/fpls.2024.1369976

**Published:** 2024-03-19

**Authors:** Changsu Lee, Sang-Il Han, Ho Na, Zun Kim, Joon Woo Ahn, Byeolnim Oh, Hyun Soo Kim

**Affiliations:** ^1^ Bio Division, NCell. Co., Ltd., Seoul, Republic of Korea; ^2^ Advanced Radiation Technology Institute, Korea Atomic Energy Research Institute, Jeongeup, Republic of Korea; ^3^ Department of Electronic Engineering, Kwangwoon University, Seoul, Republic of Korea

**Keywords:** *Arthrospira platensis*, whole genome sequencing, cell size, harvest, microfluidic

## Abstract

**Introduction:**

Cyanobacteria are typically of a size that can be observed under a microscope. Here, we present cyanobacteria of a size that can be observed with the naked eye. *Arthrospira platensis* NCB002 strain showed differentiated morphological characteristics compared to previously reported *Arthrospira* spp.

**Methods:**

*Arthrospira platensis* NCB002 was obtained by the UV irradiation of *Arthrospira* sp. NCB001, which was isolated from freshwater and owned by NCell Co., Ltd. *A. platensis* NIES-39 was obtained from the National Institute for Environmental Studies (Tsukuba, Japan). We used various analytical techniques to determine its overall characteristics.

**Results and discussion:**

The draft genome of strain NCB002 consists of five contigs comprising 6,864,973 bp with a G+C content of 44.3 mol%. The strain NCB002 had an average length of 11.69 ± 1.35 mm and a maximum of 15.15 mm, which is 23.4–50.5 times longer than the length (0.3–0.5 mm) of previously known *Arthrospira* spp., allowing it to be harvested using a thin sieve. Transcriptome analysis revealed that these morphological differences resulted from changes in cell wall formation mechanisms and increased cell division. Our results show that NCB002 has outstanding industrial value and provides a comprehensive understanding of it.

## Introduction

Cyanobacteria, also known as blue-green algae, are the oldest and most ecologically pivotal photoautotrophic prokaryotes on Earth (Afreen et al., 2021). They play a key role in the global carbon/oxygen cycle (Sánchez-Baracaldo et al., 2022) and are harnessed by diverse industries as sustainable feedstock for the production of high-value compounds (Boutarfa et al., 2022; Santos-Merino et al., 2023). Moreover, they are considered ideal candidates for the development of next-generation biofuels and other biotechnologically relevant substances because of their potential for genetic manipulation and their ability to proliferate under diverse environmental conditions (Tan et al., 2015; Patel et al., 2019).

Among these, *Arthrospira* sp., colloquially known as ‘Spirulina’, is a helical filamentous cyanobacterium, and has attracted commercial interest because of its distinctive appearance and outstanding nutritional benefits (Selmi et al., 2011; Esquivel-Hernández et al., 2021; Tzachor et al., 2021). *Arthrospira* sp. contains an abundance of protein (60–70% of dry weight, DW), carbohydrates (15–25% of DW), lipids/PUFAs (3–9% of DW), and other nutrients, making it suitable as a functional food (Wu et al., 2021; AlFadhly et al., 2022). In addition, *Arthrospira* spp. contain abundant phycocyanin, a blue photosynthetic pigment utilized in the food, cosmetic, and pharmaceutical industries (Wu et al., 2021). These commercial advantages have led to the widespread cultivation of *Arthrospira* spp. Consequently, research aimed at improving the productivity of this organism is of significant value.

Numerous studies have been conducted to determine the optimal growth conditions that maximize the production of biomass and commercially valuable products from cultures of *Arthrospira* spp (Gómez et al., 2021; de Souza da Silva et al., 2022). An alternative approach involves developing mutants with increased biomass and metabolites via genetic engineering and random mutagenesis. Although genetic engineering focuses on gene expression and transcription (Wei et al., 2019), random mutagenesis begins with the phenotypes of strains (Cheng et al., 2017), thereby conferring an advantage in identifying beneficial phenotypic mutants (Cheng et al., 2019). Ultraviolet (UV) mutagenesis, a random mutagenesis method, can cause extensive DNA damage, can be applied on a large scale, and is relatively simple and cost-effective to implement (Sivaramakrishnan and Incharoensakdi, 2017). Thus, UV mutagenesis is an effective tool for developing phenotypic *Arthrospira* spp. mutants with increased biomass and metabolite production.

*Arthrospira* sp. possesses distinctive morphological characteristics that are essentially regarded as part of their identity (‘spira’ in Latin means spiral). Nonetheless, under diverse environmental conditions such as nutrient availability, salinity, temperature, and changes in the UV spectrum, *Arthrospira* sp. undergoes morphological change from a helical to a linear shape (Nübel et al., 2000; Mühling et al., 2003; Wu et al., 2005; Mühling et al., 2006; Gao et al., 2008). This morphological change is accompanied by enhanced cell flotation and biomass productivity (Yadav et al., 2020). In addition, unlike other spiral-shaped bacteria that are unicellular, *Arthrospira* spp. are multicellular and consist of cells connected to each other in a single row that elongates through cell division (Chaiyasitdhi et al., 2018). Therefore, the development of elongated linear *Arthrospira* sp. mutants could provide potential industrial advantages such as enhanced biomass productivity and simplified harvesting. However, to the best of our knowledge, there are no cases in which UV mutagenesis has been used to develop elongated linear *Arthrospira* spp. mutants.

**Figure 1:**
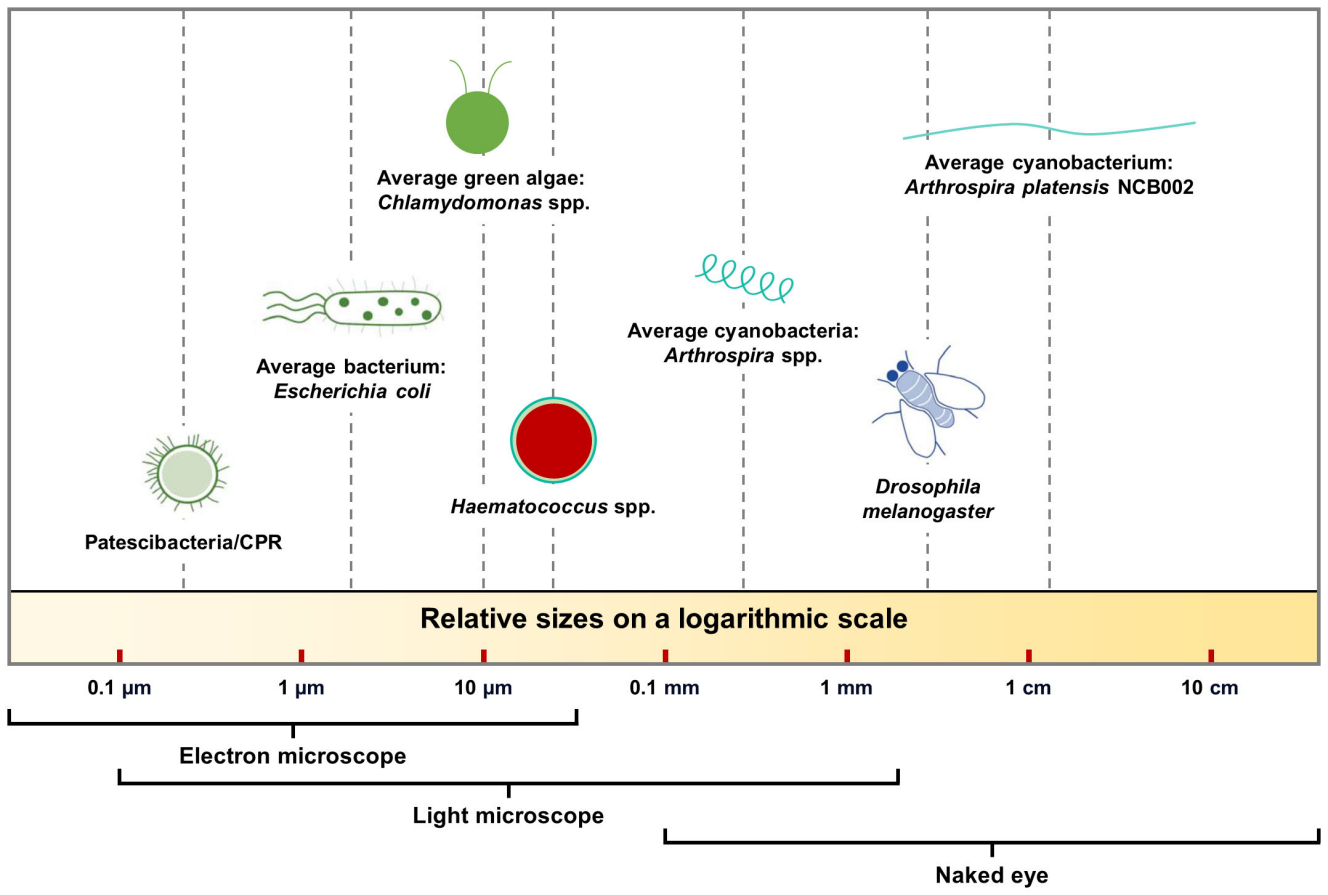
Size comparison of various prokaryotic and eukaryotic organisms on a log scale.

In this study, we developed an elongated linear *Arthrospira* sp. mutant from an indigenous Korean *Arthrospira* sp. by UV mutagenesis (**Figure 1**). The physiological characteristics and biomass productivity of the mutant strain were evaluated and compared with those of the most phylogenetically similar strain, *A. platensis* NIES-39. Furthermore, both the phenotypic and genotypic characteristics of the mutant strain were characterized. To the best of our knowledge, this is the first study to report an elongated linear *Arthrospira* spp. mutant development via UV mutagenesis. We anticipate that our research will pave the way for new commercial and ecological applications of *Arthrospira* spp.

## Materials and methods

### Microalgal strains and culture conditions

*Arthrospira platensis* NCB002 was obtained by the UV irradiation of *Arthrospira* sp. NCB001, which was isolated from freshwater and owned by NCell Co., Ltd. *A. platensis* NIES-39 was obtained from the National Institute for Environmental Studies (Tsukuba, Japan).

The inoculation densities of the two microalgal strains were 0.1 g m^-1^. First, to compare the characteristics of NCB002 with those of NIES-39, each of the two algal strains was cultured on SOT media (Ogawa, 1970) in a 1 L media bottle under aeration for 10 days. The light intensity was set at an average of 175 ± 5 *μ*mol m^2^ s^-1^ and aeration was maintained at 0.1 vvm using air with a 0.2 *μ*m syringe filter. During cultivation, the light cycle was set to 24:0 h (continuous illumination). After culturing, various analyses were performed to compare the two microalgal strains. At least three biological replicates were used to experiments.

### Characteristics of the microalgal *Arthrosprira platensis*

#### Dry weight

To determine the dry weight, 10 mL of the culture solution was filtered through Whatman GF/C ^TM^ glass microfiber filter paper (1.2 *μ*m pore size; Cytiva, Marlborough, USA) and incubated at 105^°C^C for 24 h in a drying oven. The dried filters were cooled at 25^°C^C, and reweighed. The dry weights were calculated by subtracting the filter weight from the total weight.

#### Phycocyanin content

Cultured microalgal samples (1 mL) were centrifuged at 15,000 rpm for one minute. The cell pellet was resuspended in 1 mL of 0.05 M phosphate buffer, then extracted for 1 min using a MiniBeadBeater-16 (Model 607 EUR, Biospec, Bartlesville, USA). The extracted solution was centrifuged at 15,000 rpm for 1 min. The supernatant of the extracted solution was analyzed using a UV-VIS spectrophotometer (Lambda 365, PerkinElmer, Waltham, MA), using the phycocyanin equation (Bennett and Bogorad, 1973).


$$\text{Phycocyanin content}=({\text{OD}}_{615\text{nm}}–0.474{\text{ OD}}_{652\text{nm}})/5.34$$

#### Chlorophyll content

Cultured microalgal samples (1 mL) were centrifuged at 15,000 rpm for 1 min. The cell pellet was resuspended in 1 mL of 90% acetone, then extracted for 1 min using a MiniBeadBeater-16 (Model 607 EUR, Biospec, Bartlesville, USA). The extracted solution was centrifuged at 15,000 rpm for 1 min. The supernatant of the extracted solution was measured using a UV-VIS spectrophotometer and Chl equations (Li et al., 2000; Vo et al., 2016).


$$\text{Chl a}=12.21{\text{ OD}}_{663\text{nm}}–2.81{\text{ OD}}_{646\text{nm}}$$

$$\text{Chl b}=20.13{\text{ OD}}_{646\text{nm}}–5.03{\text{ OD}}_{663\text{nm}}$$

Total Chl=Chl a+Chl b

### Analysis of nutrient composition

To analyze the amino acid content, 18 components were analyzed using the ninhydrin post-column reaction method with ion exchange chromatography by modifying the AOAC method (Peace and Gilani, 2005). In the case of 16 amino acid components, 0.2 g of the sample was placed in a decomposition tube, 10 ml of 6 N HCl was added, nitrogen gas was injected, and the sample was hydrolyzed at 110°C for 24 hours. The filtrate was concentrated using a vacuum concentrator, adjusted to 50 mL with 0.2 M sodium citrate buffer, and then filtered through a 0.20 *μ*m cellulose acetate syringe filter and used as an analysis sample. The performic acid oxidation method was used for the sulfur-containing series of methionine and cysteine, whereas the alkaline hydrolysis method was used for tryptophan.

### RNA isolation, library preparation, and sequencing

Harvested *A. platensis* NCB002 was frozen in liquid nitrogen and crushed using a mortar and pestle. Total RNA was isolated using TRIzol reagent (Invitrogen, USA). RNA quality was evaluated using an Agilent 2100 bioanalyzer with an RNA 6000 Nano Chip (Agilent Technologies, Netherlands), and its concentration was determined using an ND-2000 Spectrophotometer (Thermo Inc., USA). For both control and test RNAs, rRNA was depleted using the RiboCop rRNA Depletion for Bacteria Probe Mix G+/– kit (LEXOGEN, Inc., Austria) from 1 *μ*g of total RNA. The RNA library was constructed using the NEBNext Ultra II Directional RNA-seq kit (New England Biolabs, Inc., UK) according to the manufacturer’s instructions. The rRNA-depleted RNAs were used for cDNA synthesis and shearing following the manufacturer’s instructions. Indexing was performed using Illumina indexes 1–12. The enrichment step was performed through PCR. Subsequently, the libraries were checked using a TapeStation HS D1000 ScreenTape (Agilent Technologies, Netherlands) to evaluate the mean fragment size. Quantification was performed using a library quantification kit and StepOne Real-Time PCR System (Life Technologies, Inc., USA). High-throughput sequencing was performed using paired-end 100 sequencing on a NovaSeq 6000 platform (Illumina, Inc., USA).

### Data analysis of RNA sequencing

The RNA-seq reads from strain NCB002 were mapped using the Bowtie2 software tool to obtain an alignment file. Differentially expressed genes (DEGs) were determined based on counts from both unique and multiple alignments using the EdgeR package (Gentleman et al., 2004) implemented in R. An alignment file was used for transcript assembly. Raw read counts were normalized using the trimmed mean of M-values and counts per million reads mapped (CPM) methods. Subsequently, p-values were calculated using Student’s t-test and ANOVA for comparison between samples. Biological functions and associated pathways were analyzed based on information available in the QuickGO (Gene Ontology and GO Annotations, https://www.ebi.ac.uk/QuickGO/) and KEGG databases (https://www.genome.jp/kegg/). Key genes presumed to be involved in cell appearance were analyzed using information provided by the National Center for Biotechnology Information (https://www.ncbi.nlm.nih.gov/).

## Results

### Phylogenetic and general genomic features of *Arthrospira platensis* NCB002

**Figure 2:**
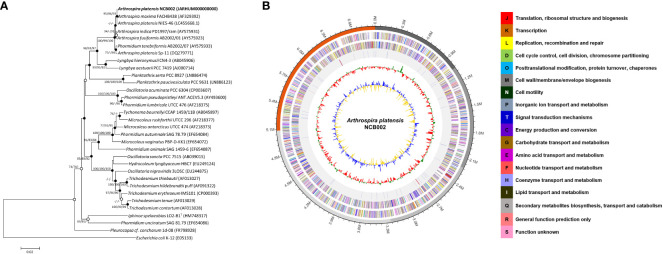
Genome map and phylogenetic tree of *Arthrospira platensis* NCB002. **(A)** Phylogenetic tree of *Arthrospira platensis* NCB002 and related species within the genus *Arthrospira* constructed using the neighbor-joining (NJ)/maximum likelihood (ML)/maximum parsimony (MP) algorithms based on 16S rRNA gene sequences. The closed circles represent nodes identified using both the ML and MP algorithms, while open circles represent nodes identified using either ML or MP algorithms. *Escherichia coli* K-12 was used as an outgroup. Bar: 0.02 accumulated changes per nucleotide. **(B)** Graphic circular map of the *Arthrospira platensis* NCB002 genome. The outer circle shows RNA genes (red, tRNA; blue, rRNA) and genes on the sense and antisense strands (colored according to COG categories), shown from the outside of the circle to the center. The inner circle shows the GC skew, with yellow and blue indicating positive and negative values, respectively; the GC content is indicated in red and green. This genome map was visualized using EzBioCloud (CJ Bioscience, Inc.).

*Arthrospira* sp. strain, designated NCB002, was isolated by inducing random mutations via UV irradiation. To confirm the phylogenetic similarity of the strain, a 16S rRNA gene-based phylogenetic analysis was conducted on strain NCB002, which was shown to be 99.86% similar to *A. platensis* NIES-46 (**Figure 2A**). The genome of *A. platensis* NCB002 was 6,864,973 bp long with a G+C content of 44.3 mol% (**Supplementary Table 1**). The draft genome of strain NCB002 was predicted to contain 5 contigs of 1,039,212 bp, 1,169,927 bp, 1,047,132 bp, 1,829,855 bp, and 1,778,847 bp. The genome of the NCB002 strain contained 6,503 coding sequences, six rRNA genes (two of the 16S-5S-23S RNA gene operon), and 12 tRNA genes (**Figure 2B**). Functional analysis was performed using the Cluster of Orthologous Groups (COG) database (http://www.ncbi.nlm.nih.gov/COG/) to analyze the genome of strain NCB002 and annotate 5,259 genes. The annotated gene was composed of the following categories: function unknown (S; 2347 genes), replication, recombination and repair (L; 665), signal transduction mechanisms (T; 364 genes), cell wall/membrane/envelope biogenesis (M; 228 genes), energy production and conversion (C; 207 genes), amino acid transport and metabolism (E; 201 genes), posttranslational modification, protein turnover, chaperones (O; 175 genes), defense mechanisms (V; 172 genes), translation, ribosomal structure and biogenesis (J; 150 genes), inorganic ion transport and metabolism (P; 147 genes), coenzyme transport and metabolism (H; 125 genes), and carbohydrate transport and metabolism (G; 124 genes) (**Supplementary Table 2**). In addition, SEED Viewer version 2.0 confirmed that more than 7% of the major categories included genes required for “RNA metabolism” (449 genes), “amino acids and derivatives” (199 genes), “carbohydrates” (171 genes), “cofactors, vitamins, prosthetic groups, pigments” (149 genes), “protein metabolism” (134 genes), and “DNA metabolism” (132 genes) (**Supplementary Figure 1**).

### Comparative genomic analysis of *Arthrospira platensis* NCB002

**Figure 3:**
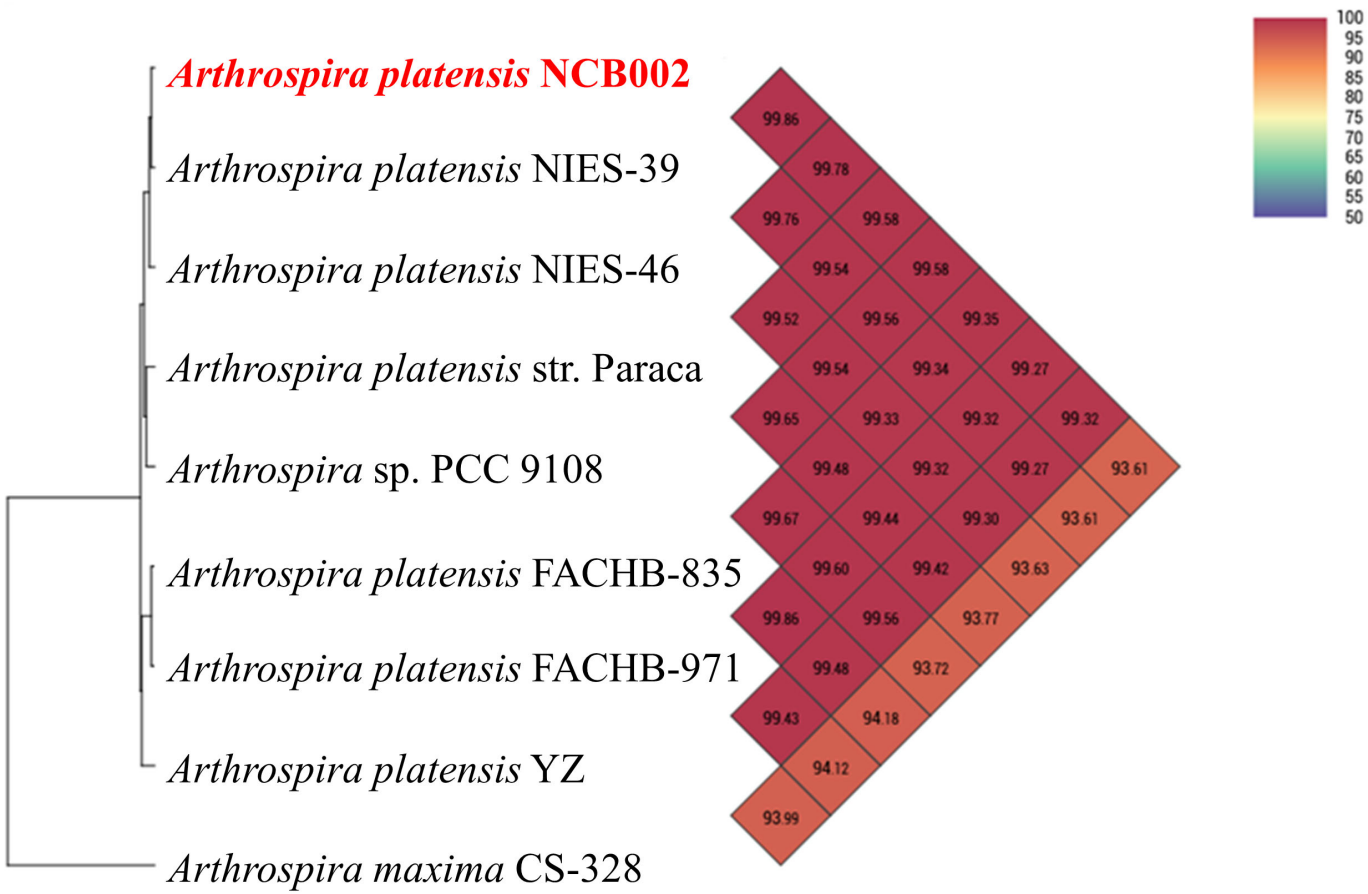
Heatmap and phylogenetic tree of *Arthrospira platensis* NCB002 generated with OrthoANI value.

The orthologous average nucleotide identity (OrthoANI) value of the *Arthrospira* genome sequence showed that strain NCB002 had 93.61–99.86% genome sequence similarity with other species and subspecies. Its genome was the most similar to that of *A. platensis* NIES-39 (99.86%), followed by *A. platensis* NIES-46 (99.78%), *A. platensis* str. Paraca (99.58%), *A.* sp. PCC 9108 (99.58%), *A. platensis* FACHB-835 (99.35%), *A. platensis* YZ (99.32%), *A. platensis* FACHB-971 (99.27%), and *A. maxima* CS-328 (93.61%; **Figure 3**). Through comparative whole-genome sequence analysis, the strain NCB002 was confirmed to belong to *A. platensis*. Venn diagrams were analyzed based on the Pan-genome Orthologous Groups (POGs) of strain NCB002 and closely related strains, and 3,821 shared and 58 unique genes were identified (**Supplementary Figure 2**).

### Nutritional value of *Arthrospira platensis* NCB002

Nutritional component analysis of three major nutrient groups (carbohydrates, proteins, and lipids), vitamins, minerals, fatty acids, and amino acids, was performed on the dry powder of *A. platensis* NCB002 harvested after culturing for one week. The three major nutritional components of NCB002 were carbohydrates (15.4%), proteins (66.7%), and lipids (5.4%) (data not shown). These results were 1.8% higher for carbohydrates, within the normal range for protein, and 0.6–2.3% lower in fat than the general *Arthrospira* spp. strains. The total lipid composition of strain NCB002 was subjected to fatty acid methyl ester analysis and compared with that of general *Arthrospira* spp. strains. Linoleic acid (C 18:1; LA) and gamma-linoleic acid (C 18:3; GLA), which are essential fatty acids, were in the common ranges of 11.45% and 19.96%, respectively. Lauric acid, stearic acid, and behenic acid, which are saturated fatty acids, were low or not detected at 0.10%, 0.89%, and 0.00%, respectively (**Table 1**). Palmitoleic acid (C 16:1), an unsaturated fatty acid, was 0.83–4.11% higher than that previously reported for other *Arthrospira* spp. strains.

**Table 1:** Comparison of fatty acid composition in *Arthrospira* spp. (Pascaud, 1993; Ötleş and Pire, 2001; Tokuşoglu and Üunal, 2003; Bensehaila et al., 2015; Ragaza et al., 2020).

Fatty acids	Nomenclature	General *Arthrospira* spp.	*Arthrospira platensis* NCB002
Lauric acid	C 12:0	0.84–3.10%	0.10%
Myristic acid	C 14:0	0.2–3.60%	0.28%
Palmitic acid	C 16:0	25.0–43.65%	33.69%
Palmitoleic acid (ω - 6)	C 16:1	0.52–3.8%	4.63%
Stearic acid	C 18:0	0.95–8.82%	0.89%
Oleic acid (ω - 6)	C 18:1	0.33–16.6%	5.46%
Linoleic acid (ω - 6)	C 18:2	9.43–17.19%	11.45%
Gamma linolenic acid (ω - 6)	C 18:3	3.64–40.1%	19.96%
Behenic acid	C 22:0	Trace–20.01%	ND

*ND, No detection.

The vitamin and mineral levels of strain NCB002 are as follows: vitamin B1 (Thiamine), 0.3 mg 100 g^-1^; vitamin B2 (Riboflavin), 4.05 mg 100 g^-1^; vitamin B12 (cyanocobalamin), 0.74 *μ*g 100 g^-1^; vitamin B6 (Pyridoxine), 0.11 mg 100 g^-1^; vitamin C, 10.45 mg 100 g^-1^; vitamin K, 12.48 mg 100 g^-1^; zinc (Zn), 4.23 mg 100 g^-1^; phosphorous (P), 1494.69 mg 100 g^-1^; iron (Fe), 115.04 mg 100 g^-1^; sodium (Na), 722.56 mg 100 g^-1^ (data not shown). In particular, vitamin K and zinc showed 11.39–11.45-fold and 1.17–2.92-fold higher values, respectively, compared to *Arthrospira* spp. strains (Ragaza et al., 2020).

The total amino acid composition of strain NCB002 was analyzed by high-performance liquid chromatography (HPLC). Among the essential amino acids, the content of leucine (5,730 mg 100 g^-1^), lysine (3,160 mg 100 g^-1^), methionine (1,460 mg 100 g^-1^), phenylalanine (2,930 mg 100 g^-1^), and threonine (3,310 mg 100 g^-1^) was higher than those of the average *Arthrospira* spp. strains (**Table 2**). Among the non-essential amino acids, the contents of aspartic acid (6,060 mg 100 g^-1^), cysteine (750 mg 100 g^-1^), glycine (3,350 mg 100 g^-1^), histidine (1,020 mg 100 g^-1^), proline (2,510 mg 100 g^-1^), serine (3,200 mg 100 g^-1^), and tyrosine (2,600 mg 100 g^-1^) were higher than those of the *Arthrospira* spp. strain.

**Table 2:** Comparison of amino acid composition in *Arthrospira* spp. (Dillon, 1995; Volkmann et al., 2008; Sharoba, 2014; Salmeán et al., 2015; Ragaza et al., 2020).

Amino acids	General *Arthrospira* spp.	*Arthrospira platensis* NCB002
	(mg per 100 g dry mass)*	(mg per 100 g dry mass)*
Essential amino acids		
Isoleucine	3500	3350
Leucine	5380–5400	5730
Lysine	2900–2960	3160
Methionine	1170–1400	1460
Phenylalanine	2750–2800	2930
Threonine	2860–3200	3310
Tryptophan	900–1090	700
Valine	3940–4000	3810
Non-essential amino acids		
Alanine	470–4590	
Arginine	430–4310	4190
Aspartic acid	610–5990	6060
Cystine	60–590	750
Glutamic acid	910–9130	8710
Glycine	320–3130	3350
Histidine	100–1000	1020
Proline	270–2380	2510
Serine	320–2760	3200
Tyrosine	300–2500	2600

### Morphological features of *Arthrospira platensis* NCB002

**Figure 4:**
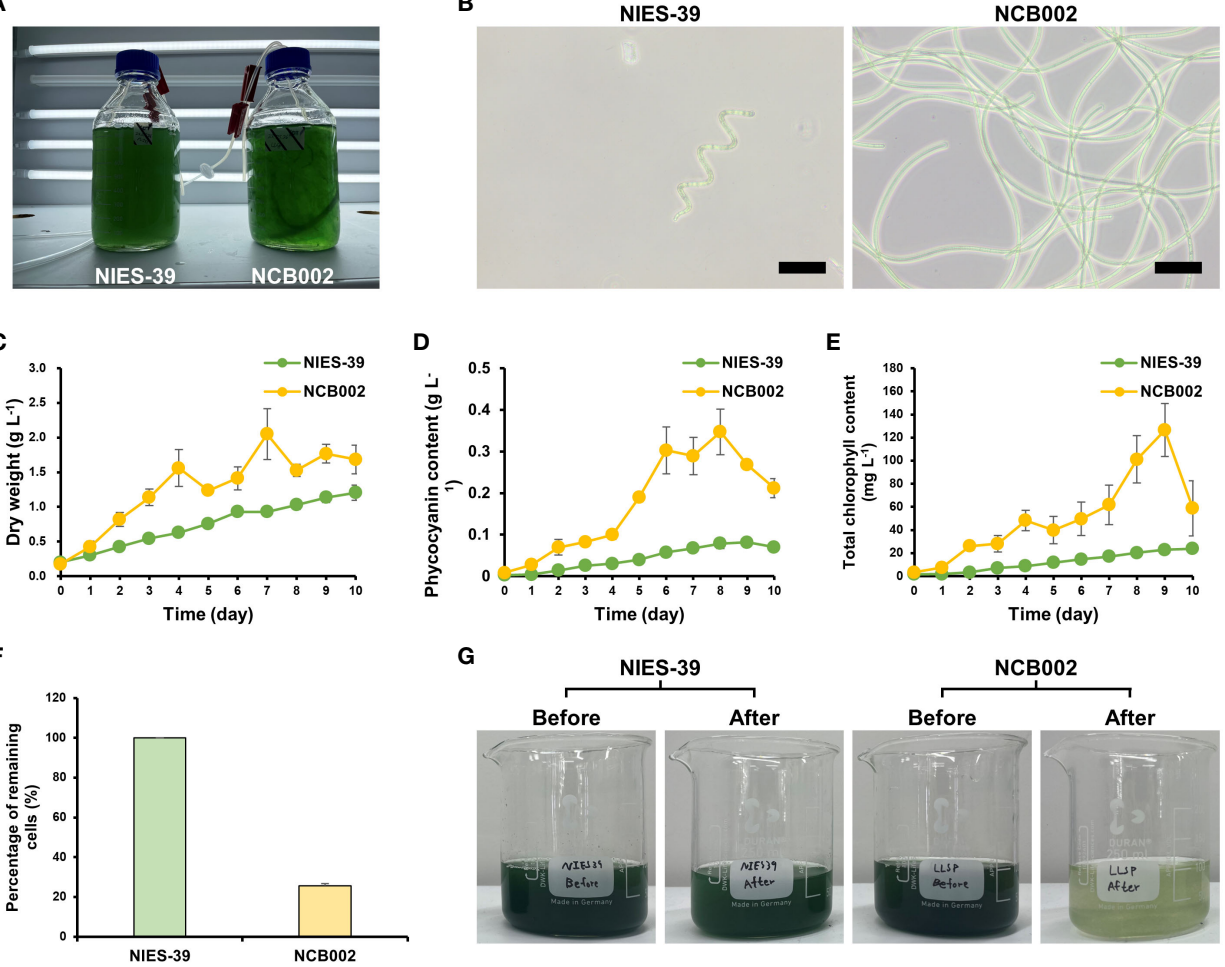
Morphological characterization of *Arthrospira platensis* NCB002. **(A)** Morphology of growth culture, **(B)** microscopic comparison of strain NIES-39 and NCB002, **(C)** dry cell weight (DCW), **(D)** phycocyanin, **(E)** chlorophyll content, **(F)** percentage of remaining cells after harvest, **(G)** comparison before and after harvest.

To determine the morphological characteristics of strain NCB002, a comparative experiment was conducted with strain NIES-39, which is most phylogenetically similar to *A. platensis*. During the general growth culture, strain NCB002 can be seen to be much longer, to the extent that the two strains can be easily distinguished with the naked eye. Strain NCB002 appeared as long strands of threads (**Figure 4A**). These morphological features were clearly distinguishable under a microscope. Strain NIES-39 was observed to have a screw-like coil, whereas strain NCB002 was observed to have a long linear form (**Figure 4B**). During the growth process of the two strains, strain NCB002 had a higher average biomass, reaching a maximum of 2.2 times higher on the 7^th^ day of cultivation (**Figure 4C**). These characteristics were also evident in the pigments of the two strains, with phycocyanin showing the greatest difference on the 8^th^ day of culture and chlorophyll showing the greatest difference on the 9^th^ day of culture (**Figures 4D, E**). Comparative analysis was performed when the two strains were harvested from the sieve on the 10^th^ day of culture. Strain NIES-39 was not harvested, with 100% of the cells remaining after harvest, whereas most cells of NCB002 were harvested through the sieve, leaving only 20% of the cells remaining (**Figures 4F, G**). To check whether there were morphological characteristics inside the cells, they were compared using transmission electron microscopy (TEM). However, no special characteristics were confirmed (**Supplementary Figure 3**).

### Length analysis of *Arthrospira platensis* NCB002 by using a microfluidic device

**Figure 5:**
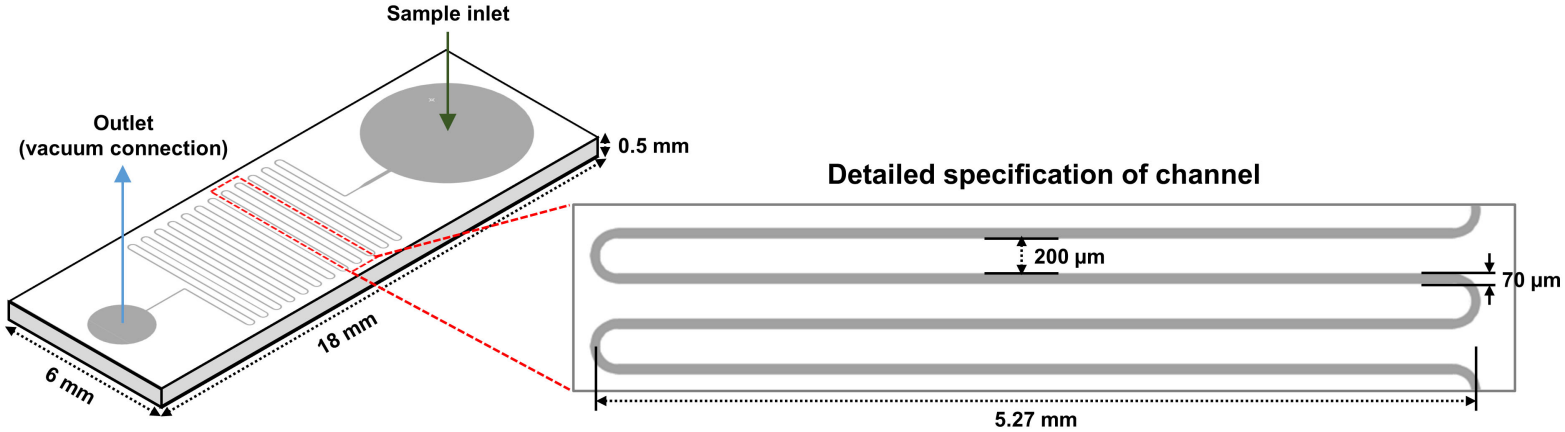
Design of a microfluidic device.

**Figure 6:**
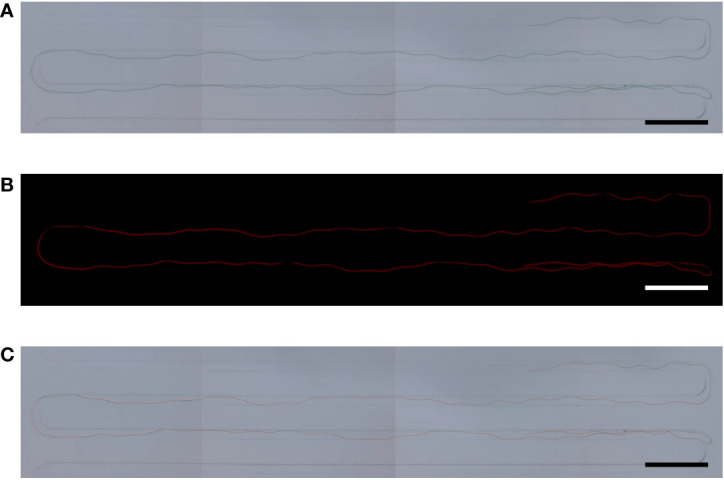
Cell size of *Arthrospira platensis* NCB002. **(A)** Microscopic image under optical light, **(B)** Microscopic image under fluorescent lamp, **(C)** Merged.

In a previous study, it was proven that the strain NCB002 is efficient for harvesting because of its long linear form. However, it was difficult to precisely determine how long it was. A microfluidic device was used to accurately measure the length of strain NCB002. The microfluidic device was designed to pass through a thin, long channel between the sample inlet and outlet. The channels were 5.27 mm wide, the distance between channels was 200 *μ*m, and the channel passage was 70 *μ*m (**Figure 5**). Strain NCB002 was passed through the manufactured microfluidic device more than 100 times, and only the cell line that passed through the channel was selected and measured using by ZEN blue program more than 30 times. The average length of strain NCB002 that passed through the channel was 11.69 ± 1.35 mm, and the maximum was 15.15 mm (**Figures 6A–C**).

### Functional enrichment and KEGG pathway analysis

**Figure 7:**
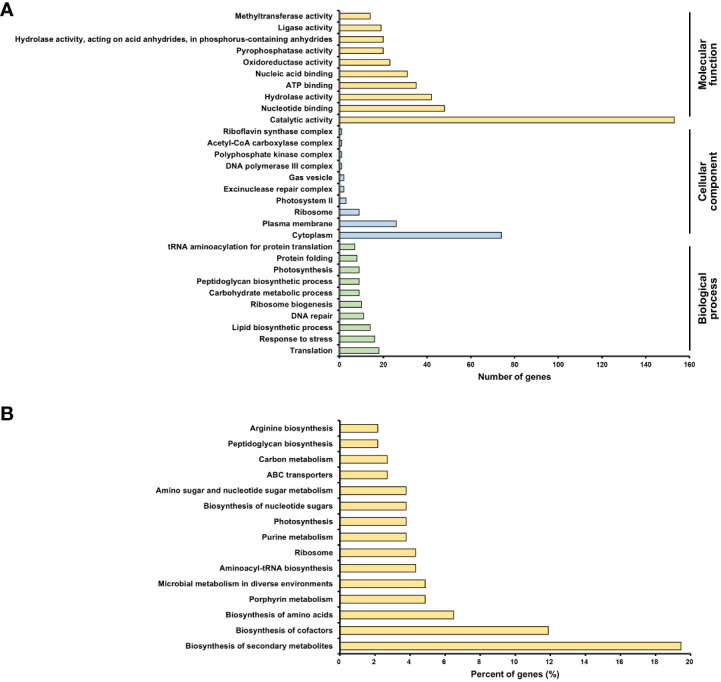
Transcriptomic analysis of *Arthrospira platensis* NCB002. **(A)** Gene ontology (GO) enrichment analysis. An overview of the top 10 significantly enriched terms in three categories: biological process, cellular component, and molecular function, **(B)** KEGG pathway enrichment analysis. The top 15 pathways were ranked based on the number of involved genes.

To evaluate differentially expressed genes (DEGs), the fold-change threshold cutoff was set at > 2-fold for increased accumulation and< 0.5-fold for decreased accumulation. Of the 1616 DEGs, 193 recognized gene symbols were used for evaluation. To obtain a biological view of the 193 DEGs, enrichment of biological process (BP), cellular component (CC), and molecular function (MF) categories were analyzed using the DAVID homology tool based on Gene ontology (GO) analysis. The majority of DEGs were classified as MF or CC based on the GO classification criteria. The largest number of DEGs was found in functional groups with catalytic activity. The cytoplasmic group accounted for the second-highest number of DEGs (**Figure 7A**).

The Kyoto Encyclopedia of Genes and Genomes (KEGG) pathway analysis revealed 15 significant pathways (P< 0.05). The top 15 pathways were biosynthesis of secondary metabolites (arp01110), biosynthesis of cofactors (arp01240), biosynthesis of amino acids (arp01230), porphyrin metabolism (arp00860), microbial metabolism in diverse environments (arp01120), aminoacyl-tRNA biosynthesis (arp00970), ribosome (arp03010), purine metabolism (arp00230), photosynthesis (arp00195), biosynthesis of nucleotide sugars (arp01250), amino sugar and nucleotide sugar metabolism (arp00520), ABC transporters (arp02010), carbon metabolism (arp01200), peptidoglycan biosynthesis (arp00550), and arginine biosynthesis (arp00220) (**Figure 7B**).

**Figure 8:**
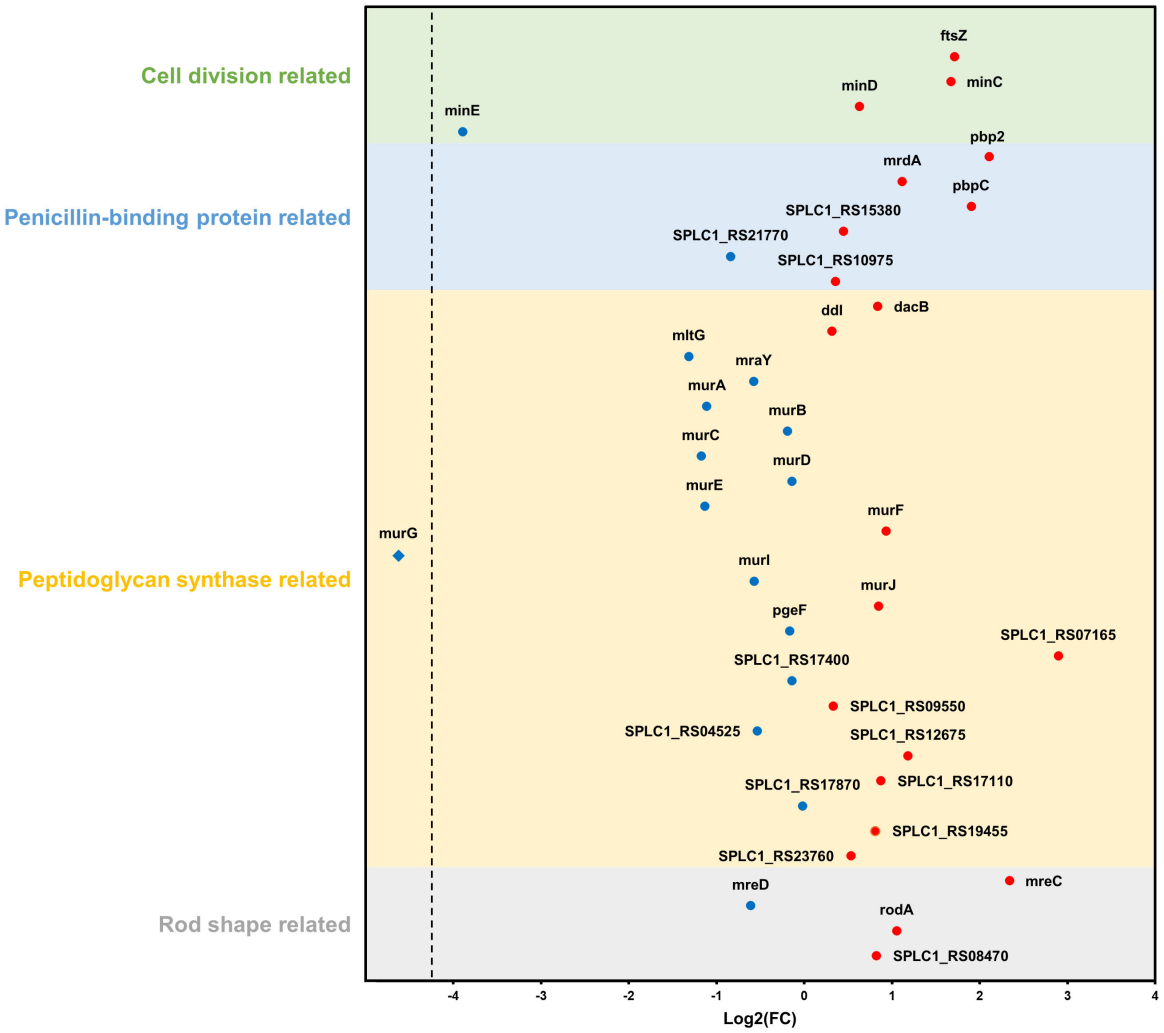
Changes in gene expression relate to four categories: cell division, penicillin-binding protein, peptidoglycan synthase, and rod shape. Blue and red dots indicate downregulation and upregulation in the strain NCB002 than in the strain NIES-39, respectively. The blue rhombus (murG) indicates no expression in the strain NCB002.

Furthermore, based on the NCBI database, additional analysis was performed on DEGs belonging to four categories (cell division-, penicillin-binding protein-, peptidoglycan synthase-, and rod shape-related genes) that were expected to be particularly correlated with phenotypic features. Among the 23 genes categorized as peptidoglycan synthase-related, two genes (SPLC1_RS07165 and SPLC1_RS12675) were upregulated and five genes (mltG, murA, murC, murE, and murG) were downregulated. Of the four genes associated with rod-shape determination, mreC and rodA were upregulated. Among the six genes identified to be related to penicillin-binding proteins (PBP), three genes, pbp2, mrdA, and pbpC, were upregulated. Finally, among the four genes linked to cell division, ftsZ and minC were upregulated, whereas minE was downregulated (**Figure 8**).

## Discussion

In general, the harvest stage accounts for 20–30% of the total production cost of microalgae (Grima et al., 2003; Mata et al., 2010; Verma et al., 2010), and various technologies are being researched to improve harvesting methods (Singh and Patidar, 2018). Our research focused on strain improvement and the induction of random mutations through UV irradiation. Strain NCB002, newly obtained through UV irradiation, is dramatically longer than previously known *Arthrospira* spp. strains. Based on these characteristics, strain NCB002 has a significant advantage in the harvesting process. When harvested through a thin sieve, almost 80% of strain NCB002 was recovered, whereas NIES-39, which was the reference strain, was not recovered at all through this sieving process (**Figures 4F, G**). Strain NCB002 can be washed with a sieve because of its length, which makes sieving effective for removing impurities from the culture. The cell culture-related characteristics of this strain include a high growth rate and high phycocyanin and chlorophyll contents (**Figures 4C–E**). The increase in the content of the main pigments, phycocyanin and chlorophyll, which are major high-value-added substances, appears to be due to the increase in biomass.

Here, we conducted genomic, transcriptomic, microfluidic technology, and TEM analysis to obtain a comprehensive understanding of this strain and sought to identify any differences compared to existing *Arthrospira* spp. strains. Strain NCB002 has a long linear form, which is different from the helical form of *A. platensis* (**Figure 4B**). These morphological characteristics significantly increased the harvest efficiency (**Figure 4F**). However, it was difficult to measure the exact length of this strain in the single-cell state, although its elongated appearance could be observed with the naked eye. In General, the length of a single cell of *Arhrospira* spp. is 50–500 *μ*m in diameter and 3–4 *μ*m in width and can only be measured using a microscope (Vo et al., 2015). However, strain NCB002 was so long that it was difficult to measure it even under a microscope. A microfluidic device was used to measure the length of strain NCB002 more accurately (**Figure 5**). This device could not measure cells that were excessively long because they broke or agglomerated during passage. Therefore, the current length was measured based on the cells that passed through this device, showing an average length of 11.69 ± 1.35 mm and a maximum length of 15.15 mm (**Figures 6A–C**). Transmission electron microscopy (TEM) was performed to confirm the differences within the cells; however, no notable differences were identified (**Supplementary Figure 3**).

Whole genome sequencing (WGS) confirmed that this strain belonged to *Arthrospira platensis* phylogenetically at 99.86%, with a total genome size of 6,864,973 bp and a G + C content of 44.3 mol% (**Figures 2B**, **3**). Compared with existing reference strains, it was confirmed that it had 3,821 core genes and 58 unique genes (**Supplementary Figure 2**).

To determine why strain NCB002 was longer than the existing strains, a transcriptomics analysis was conducted. GO enrichment analysis was conducted to categorize differentially expressed genes (DEGs) (**Figure 7A**). Genes within the catalytic activity functional group are primarily associated with proteins that play catalytic roles in diverse chemical reactions, particularly enzymes that are pivotal to cellular metabolic pathways. Therefore, the differential expression of genes related to catalytic activity indicates potential metabolic or enzymatic process modifications in the NCB002 strain. In strain NCB002, 72 genes linked to catalytic function were overexpressed, suggesting a possible enhancement or reduction in metabolic activity. This can influence nutrient uptake, growth kinetics, and the production of specific metabolites in cells. The cytoplasm contains proteins and cellular components that are integral to the cytoplasm, which is the primary internal cellular matrix. It is associated with fundamental cellular functions and elements involved in a range of biological processes, including metabolism, signaling pathways, and cell division. Therefore, differential expression of cytoplasm-linked genes suggests potential alterations in cell organization, intracellular transport, and cellular homeostasis in NCB002 cells. Given that *Arthrospira* sp. exhibits a filamentous structure connecting its multicellular organization, alterations in intercellular signaling, transport mechanisms, cell division machinery, and cell-to-cell adhesion may have contributed to the enhancement of the chain length.

KEGG pathway enrichment analysis revealed alterations in metabolic pathways, metabolite production, intracellular transport, and cytoplasmic machinery responsible for cell division and intercellular adhesion (**Figure 7B**). Notably, pathways for the biosynthesis of secondary metabolites and cofactors were predominant. These pathways may facilitate the formation or maintenance of long chains by amplifying the production of specific molecules that are instrumental in stress responses, signaling, or cell-to-cell interactions. The observed changes in pathways related to amino acid biosynthesis, purine metabolism, nucleotide sugar biosynthesis, and carbon metabolism indicate the occurrence of a shift in the metabolic landscape of NCB002. These changes may have been accompanied by changes in nutrient assimilation, energy utilization, and growth in NCB002. Additionally, the ABC transporter pathway suggests changes in nutrient uptake and transport in NCB002. Nutrient uptake mechanisms and nutrient or molecular transport may have been enhanced or diversified in NCB002. The peptidoglycan biosynthesis pathway is likely linked to modifications in the cytoplasmic machinery that governs cell division and intercellular adhesion. Furthermore, pathways such as aminoacyl-tRNA biosynthesis, ribosome biosynthesis, and arginine biosynthesis implied potential changes in the protein expression landscape of NCB002. The photosynthetic pathway suggests potential modifications in light capture or energy conversion, which may affect both energy efficiency and growth rate in NCB002.

Integrating the phenotypic features of NCB002 with GO and KEGG pathway enrichment analyses, alterations in photosynthesis, nutrient uptake, nutrient assimilation, metabolic pathways, and metabolite production appear to be involved in cell division. In addition, modifications in intercellular adhesion seem to be instrumental in elongating chain formation. Furthermore, it can be inferred that changes in stress response, signal transduction, and intercellular interactions are involved in the maintenance of extended chains. In other words, it was inferred that changes in NCB002 are multifaceted, encompassing metabolic, morphological, and ecological changes. However, a deeper exploration of these pathways is necessary to elucidate the precise mechanisms driving these phenotypic features and comprehend their potential advantages or implications.

The bacterial cell shape is predominantly defined by the peptidoglycan that constitutes the cell wall (Rohs and Bernhardt, 2021). Therefore, the expansion and division of this structure play an integral role in bacterial growth and division. Peptidoglycan synthesis is regulated by the Rod complex, which includes mreB, mreC, mreD, rodA, rodZ, and PBP2 (Egan et al., 2020). Specifically, PBP2 functions as a transpeptidase essential for bacterial cell elongation (Sauvage et al., 2008) and rodA encodes a glycosyltransferase integral to the polymerization process of peptidoglycan synthesis (Sjodt et al., 2020). The genes mreC and mreD, along with mreB, form an operon central to maintaining the structural integrity of the cell wall (Ago et al., 2023). In addition, mreC interacts with PBP2 (Contreras-Martel et al., 2017), causing structural changes in PBP2 and stimulating peptidoglycan polymerization and cross-linking (Rohs et al., 2018). mreD negatively regulates the interaction between mreC and PBP2; consequently, the balance between mreC and mreD determines the activity of PBP2 (Liu et al., 2020). In NCB002, the observed upregulation of PBP2, mreC, and rodA coupled with the downregulation of mreD (**Figure 8**) may be reflected in the promotion of peptidoglycan insertion into the cell surface and cell elongation by the Rod complex. Moreover, the upregulation of the pbpC gene, which encodes a transglycosylase involved in peptidoglycan synthesis, can lead to the production of long-linked sugar chains. This strengthens intercellular bonds, facilitating the development of elongated chain structures.

Filamentous cyanobacteria grow through intercalary cell division (Corrales-Guerrero et al., 2018). The ftsZ gene is essential for bacterial cell division (Springstein et al., 2020) and drives peptidoglycan biosynthesis together with the divisome, a multiprotein complex (Wagstaff and Löwe, 2018). Previous research on *Escherichia coli* demonstrated that reduced ftsZ expression leads to elongated cells, whereas increased expression causes frequent cell division and formation of minicells (Ward Jr and Lutkenhaus, 1985). In contrast, the elongated mutant NCB002 exhibited overexpression of ftsZ (**Figure 8**), suggesting a fundamental difference in cell division mechanics between *E. coli*, a unicellular rod-shaped bacterium, and filamentous cyanobacteria of the genus *Arthrospira*. In *Arthrospira* sp., as long as their peptidoglycan walls are maintained, the promotion of intercellular cell division implies an increase in cell length. Moreover, the FtsZ-driven divisome plays a role beyond cell division, maintaining multicellularity and intercellular communication in filamentous cyanobacteria (Ramos-León et al., 2015). Nevertheless, for the augmented expression of the ftsZ gene to result in extended cell lengths, precise Z ring assembly is essential. The minC, minD, and minE genes, which are part of the mitotic timer pathway, help ftsZ to move to the appropriate location and form a Z ring. The upregulation of minC and downregulation of minE observed in NCB002 appear to control the formation time and location of the Z ring, and are inferred to have influenced the chain length extension of NCB002.

As previously mentioned, maintenance of the peptidoglycan wall is a prerequisite for the promotion of cell division, leading to the elongation of cells in *Arthrospira* sp. Generally, as the cell length increases, a greater quantity of peptidoglycan is required; therefore, genes related to peptidoglycan biosynthesis must be upregulated (Zhao et al., 2022). However, in the elongated mutant NCB002, the key genes involved in peptidoglycan synthesis, such as mltG, murA, murC, and murG, were downregulated (**Figure 8**). This downregulation is linked to a reduction in cell wall rigidity, as established in studies on *E. coli* (Zhang et al., 2018). Peptidoglycan layers undergo continuous turnover throughout the bacterial life cycle, with new monomers incorporated as old bonds are cleaved (Jacobs et al., 1994). Thus, when a new monomer is inserted into the existing peptidoglycan layer and cross-linked with the original layer, structural changes may occur in the peptidoglycan layer. Therefore, we propose that decreased cell wall rigidity in NCB002 cells may enhance the frequency of new monomer insertions, leading to cellular elongation. Indeed, the upregulation of genes associated with the Rod complex, which is responsible for the polymerization and cross-linking of peptidoglycans, the insertion of monomers, and the production of long glycan chains, supports this hypothesis in NCB002. However, the mechanisms underlying the downregulation of peptidoglycan biosynthesis genes are not yet fully understood.

Collectively, the long-chain phenotype of NCB002 was characterized by significant alterations in cell morphology, cell wall assembly, and cellular division dynamics. The distinctive gene expression pattern of NCB002, characterized by the upregulation of genes related to rod shape, PBP, and cell division, along with the downregulation of genes involved in peptidoglycan synthesis, suggests a complex regulatory mechanism. This mechanism orchestrates cell-to-cell adhesion, peptidoglycan synthesis, filament elongation, and cellular division, thereby contributing to the NCB002’s unique structural and physiological attributes. However, current information may be fragmentary. The roles of individual genes can vary by interactions with other genes, which can be very complex and controlled by multiple pathways. To fully elucidate the comprehensive characteristics NCB002, which has unprecedented cell length, may require the development of a novel device that can measure cell lengths non-invasively. This endeavor may also need acquiring genetic information that exceeds the current level of knowledge about *Arthrospira* sp. Thus, to gain additional insight into these changes, further in-depth studies are required.

In conclusion, strain NCB002 of NCell Co., Ltd. obtained through UV irradiation is an attractive strain, both academically and industrially, and we hope that it will be used more industrially in the future.

## Data availability statement

All relevant data supporting the key findings of this study are available within the article and its **Supplementary Information** files, or from the corresponding author upon reasonable request. The source data are provided in this study. The draft genome sequences were deposited in the DNA databank of Japan/European Molecular Biology Laboratory/GenBank under the accession numbers JARHUM010000001-JARHUM010000005.

## Author contributions

CL: Conceptualization, Data curation, Formal analysis, Funding acquisition, Investigation, Supervision, Validation, Writing – original draft, Writing – review & editing. SH: Data curation, Writing – original draft, Writing – review & editing. HN: Methodology, Writing – original draft. ZK: Methodology, Writing – original draft. JA: Data curation, Writing – original draft. BO: Methodology, Writing – original draft. HK: Data curation, Writing – original draft.
