# Supplementary material for: Comprehensive understanding of the mutant ‘giant’ Arthrospira platensis developed via ultraviolet mutagenesis
Source: Front Plant Sci. 2024 Mar 19;15:1369976. doi: 10.3389/fpls.2024.1369976 (PMC10985164; doi:10.3389/fpls.2024.1369976)
Supplement: Supplementary file 1 [file DataSheet_1.docx]

Supplementary Material

**Comprehensive understanding of the mutant 'giant' *Arthrospira platensis* developed via ultraviolet (UV) mutagenesis**

**Changsu Lee^1,†,*^, Sang-Il Han^2,†^, Ho Na^1^, Zun Kim^1^, Joon Woo Ahn^2^, Byeolnim Oh^3^ & Hyun Soo Kim^3^**

*** Correspondence:**

Changsu Lee

lcsclick@gmail.com

# Supplementary Figures and Tables

## Supplementary Figures

## Supplementary Figures

**Supplementary Figure S1.** The annotation of *Arthrospira platensis* NCB002 was generated using Rapid Annotation using Subsystem Technology (RAST).

**
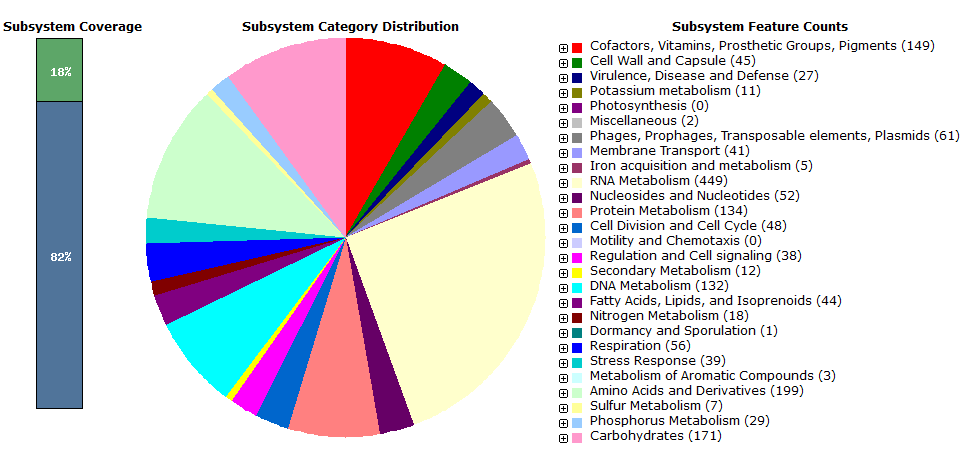
**

**Supplementary Figure S2.** Pan-genome comparisons of *Arthrospira platensis* NCB002, *A. platensis* NIES-39, *A. platensis* NIES-46, *A. platensis* str. Paraca, *A. platensis* FACHB-835, and *A. maxima* CS-328. The numbers in the center represent the core genes shared by the five strains, expressed as orthologous gene clusters in each genome.

**
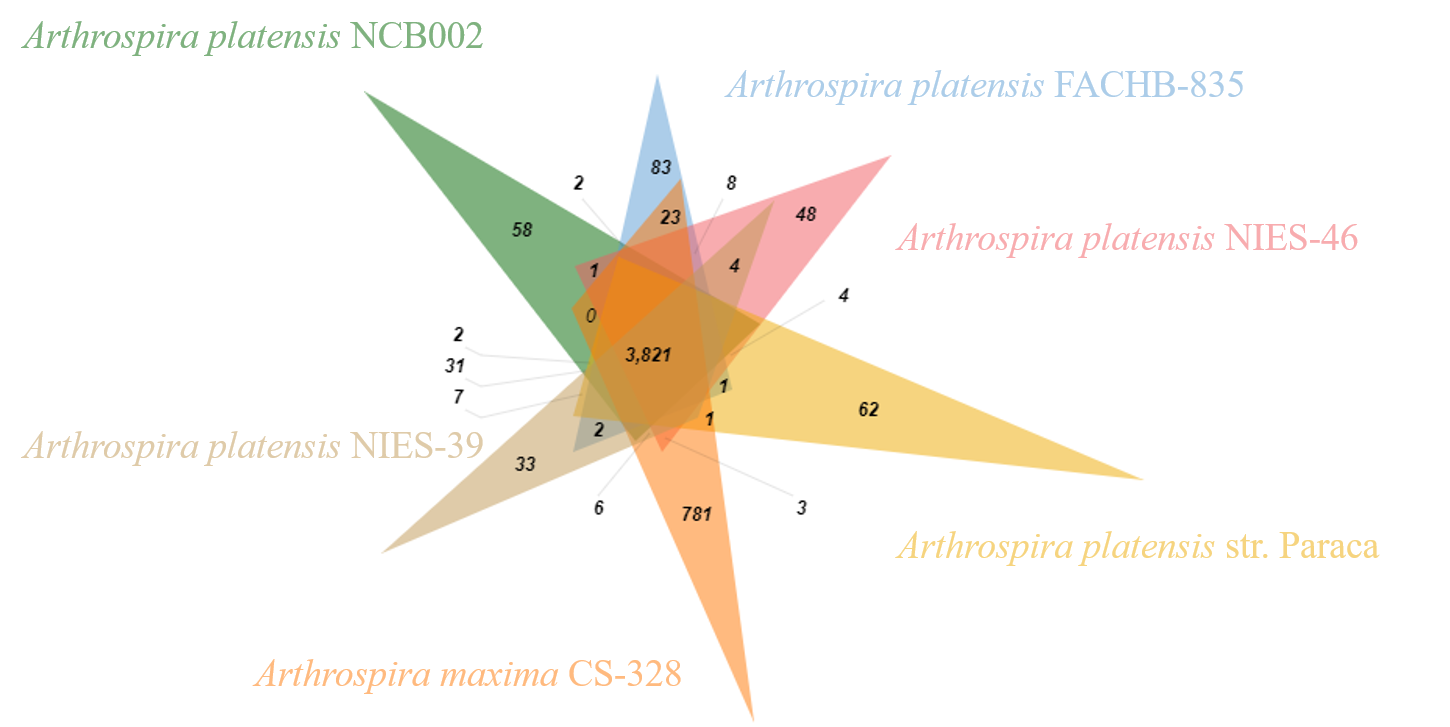
**

**Supplementary Figure S3.** Comparison of *A. platensis* NIES-39 and *A. platensis* NCB002 by transmission electron microscopy (TEM).

***A. platensis* NIES-39**

***A. platensis* NCB002**


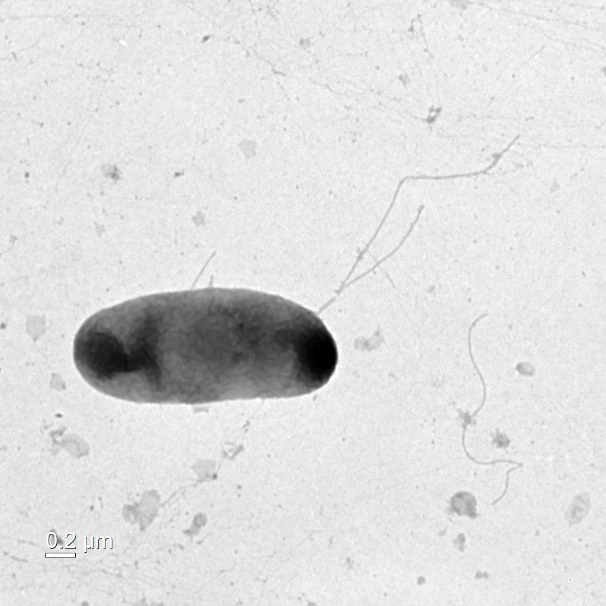

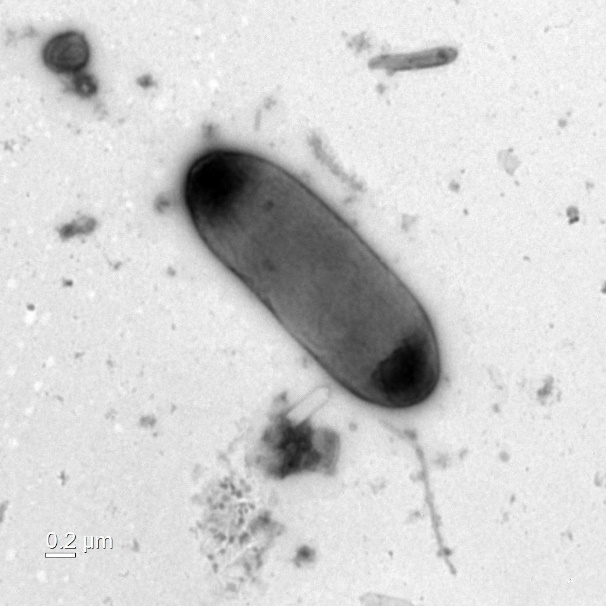


## Supplementary Tables

**Supplementary Table S1.** Feature of the *Arthrospira platensis* NCB002 genome.

|  | *Arthrospira platensis* NCB002 |
| --- | --- |
| Sequencing platforms | PacBio Sequel |
| Assembler | HGAP |
| Assembly accession | JARHUM000000000 |
| Assembly size (bp) | 6,864,973 |
| Contig numbers | 5 |
| DNA G + C content (mol%) | 44.3 |
| N50 | 1,778,847 |
| Genome coverage | 1071.0× |
| CDSs | 6,503 |
| rRNA number | 6 |
| tRNA number | 12 |

**Supplementary Table S2.** Genes involved in general COG functional categories in the genome of *Arthrospira platensis* NCB002 genome

| **COG** | | **Description** | **Number of genes** | **Percentage (%)** |
| --- | --- | --- | --- | --- |
| C | Energy production and conversion | | 207 | 3.9 |
| D | Cell cycle control, cell division, chromosome partitioning | | 28 | 0.5 |
| E | Amino acid transport and metabolism | | 201 | 3.8 |
| F | Nucleotide transport and metabolism | | 68 | 1.3 |
| G | Carbohydrate transport and metabolism | | 124 | 2.4 |
| H | Coenzyme transport and metabolism | | 125 | 2.4 |
| I | Lipid transport and metabolism | | 51 | 1.0 |
| J | Translation, ribosomal structure and biogenesis | | 150 | 2.9 |
| K | Transcription | | 86 | 1.6 |
| L | Replication, recombination and repair | | 665 | 12.6 |
| M | Cell wall/membrane/envelope biogenesis | | 228 | 4.3 |
| N | Cell motility | | 12 | 0.2 |
| O | Posttranslational modification, protein turnover, chaperones | | 175 | 3.3 |
| P | Inorganic ion transport and metabolism | | 147 | 2.8 |
| Q | Secondary metabolites biosynthesis, transport and catabolism | | 62 | 1.2 |
| R | General function prediction only | | 0 | 0.0 |
| S | Function unknown | | 2347 | 44.6 |
| T | Signal transduction mechanisms | | 364 | 6.9 |
| U | Intracellular trafficking, secretion, and vesicular transport | | 47 | 0.9 |
| V | Defense mechanisms | | 172 | 3.3 |
